# Hybrid multiscale modeling and prediction of cancer cell behavior

**DOI:** 10.1371/journal.pone.0183810

**Published:** 2017-08-28

**Authors:** Mohammad Hossein Zangooei, Jafar Habibi

**Affiliations:** Department of Computer Engineering, Sharif University of Technology, Tehran, Iran; Universita degli Studi di Catania, ITALY

## Abstract

**Background:**

Understanding cancer development crossing several spatial-temporal scales is of great practical significance to better understand and treat cancers. It is difficult to tackle this challenge with pure biological means. Moreover, hybrid modeling techniques have been proposed that combine the advantages of the continuum and the discrete methods to model multiscale problems.

**Methods:**

In light of these problems, we have proposed a new hybrid vascular model to facilitate the multiscale modeling and simulation of cancer development with respect to the agent-based, cellular automata and machine learning methods. The purpose of this simulation is to create a dataset that can be used for prediction of cell phenotypes. By using a proposed Q-learning based on SVR-NSGA-II method, the cells have the capability to predict their phenotypes autonomously that is, to act on its own without external direction in response to situations it encounters.

**Results:**

Computational simulations of the model were performed in order to analyze its performance. The most striking feature of our results is that each cell can select its phenotype at each time step according to its condition. We provide evidence that the prediction of cell phenotypes is reliable.

**Conclusion:**

Our proposed model, which we term a hybrid multiscale modeling of cancer cell behavior, has the potential to combine the best features of both continuum and discrete models. The in silico results indicate that the 3D model can represent key features of cancer growth, angiogenesis, and its related micro-environment and show that the findings are in good agreement with biological tumor behavior. To the best of our knowledge, this paper is the first hybrid vascular multiscale modeling of cancer cell behavior that has the capability to predict cell phenotypes individually by a self-generated dataset.

## Introduction

Computer-based simulation and modeling (the dry-lab experimentation) are supposed to be a potential auxiliary to the traditional biological experiments for systematically considering complex systems like cancer in systems biology. Cancer evolution is a very complex procedure, involving many dissimilar phenomena, which happen at different scales. A medical doctor, bio-chemist or a biologist would probably describe the phenomena occurring during the cancer evolution using three natural points of view: the tissue level, the cellular level and the sub-cellular level. From the modeling viewpoint, a link can be approximately drawn between the description levels above and the macroscopic, mesoscopic and microscopic scales.

Furthermore, what occurs at a certain scale is toughly related to what happens at the other scales. Consequently, it is not possible to completely describe a phenomenon without taking into account others, occurring at a larger or a smaller scale.

Multiscale cancer modelers up to now have a wealth of useful, mainly scale-specific resources to mention to or base their novel research on, however they face the massive challenge of developing more realistic and more accurate predictive models. The fundamental reason is that when regarding the number of mechanisms at multiple scales, more parameters of the model and the connections between them will have to be defined, described, quantified, and adapted frequently according to data from the clinics, experiments or literature.

The multiscale nature of cancer requires modeling approaches that can handle multiple subcellular and cellular aspects acting on different time and space scales. Hybrid models provide a way to integrate both continuous and discrete variables that are used to denote concentration or density fields and individual cells, respectively [1].

The tumor has its own vascular network which comes up with access to an almost infinite supply of resources and allows illimitable growth of the tumor mass. Recently several groups have started to improve models of angiogenesis in which individual vessels form a network that delivers nutrients to the tissue.

### Modeling approach

We significantly improved our previous agent based model [2] as a hybrid multiscale one. Such model is developed for investigating cancer cell within a three-dimensional in silico microenvironment and with angiogenesis. The aim of this paper is to study, by means of a hybrid multiscale model, the growth of a heterogeneous colony composed of healthy and cancerous cell populations, as well as to study the effect of the vasculature. While in our model the cells are viewed as discrete entities (or agent), the diffusion of nutrients is treated as a continuous field. Our agent-based sub-model is able to incorporate both cell growth and complex vascular geometry at the tissue scale. This model represents internal cellular processes via differential equations. In view of angiogenesis vital role in tumor growth, our model has a 3D visualization for angiogenesis and vascular tumor growth which shows how blood flow influences the growth of healthy and cancerous cells in cancer.

While our model simulation is running a new dataset is generated. The phenotypes and the variables of cell states, constitute the features of dataset. To the best of our knowledge, this paper is the first hybrid vascular multiscale modeling of cancer cell behaviors that has the capability to predict their phenotypes autonomously.

### Main results

Our model is formulated and performed on a three-dimensional square grid that subdivides the simulation domain into lattice sites. We study a cluster of tumor cells to which nutrient is made available through a nearby blood vessel. The proposed model is useful because it allows for predictions to be made with regard to the behavior of cancer growth. The output data are the time series of the density of each type of cell and nutrients. The findings are in good agreement with biological tumor behavior.

### Paper organization

The outline of this study is as follows. In section II we briefly review discrete, continuous and hybrid modeling of cancer. In section III we discuss the proposed hybrid modeling, where the tumor is described using both continuum and discrete elements, and which is capable of connecting cell and tissue scales to provide practical as well as theoretical insight into cancer growth. We evaluate in chapters IV and V. Conclusions and future directions are described in section VI.

## Previous work

Three major types of modeling approaches currently exist in the computational cancer modeling community: discrete, continuous and hybrid approaches. Discrete models can explicitly represent individual cells in space and time and easily incorporate biological rules. In contrast, continuum approaches, by describing e.g. the entire tumor tissue as continuum medium rather than at the resolution of individual cells, are able to capture larger-scale volumetric tumor growth dynamics. Hybrid models provide a way to integrate both discrete and continuous variables that are used to represent individual cells and concentration or density fields, respectively.

### a. Discrete modeling

The previous work in this area has consisted of two main approaches: Agent-based models (lattice-free models) [3], Cellular automata (lattice-based models) [4].

Agent-based models try to address these problems by eliminating as many artificial constraints as possible. Agent Based Modeling (ABM) revolve around modeling individuals, interactions between individuals, and in some cases, interactions with a physical or influential surrounding environment [5]. Cells by arranging themselves in non-uniform alignments are capable of moving freely through an environment. The cellular automata (CA) models share common features using CA rules from cellular or subcellular levels and using stochastic methods see detail in Wolfram [6].

#### a.1. Cellular automata modeling

In brief, a cellular automaton (CA) contains a lattice of any finite number in dimensions of cells. Each CA cell has a state. The number of state possibilities is typically finite. Each CA cell has a neighborhood. This can be defined in any number of ways, but it is typically a list of adjacent cells. The two most common options in a 3D grid of squares are the *von Neumann* neighborhood, where each cell interacts only with its six horizontal and vertical adjacent mates, and the 26 *Moore* neighborhood, comprising all the immediately adjacent cells.

The main benefit of using cellular automata in cancer modeling is the ability to observe emerging population level dynamics without a-priori knowledge of tumor behavior [7]. Summary of some important published approaches based on CA is shown in **Table 1**.

**Table 1:** Summary of some important published CA models.

Ref	Modeling Scope	Vascular/Avascular	Dimension	Year
[8]	Tumor Growth	Avascular	2D	1993
[9]	Tumor Growth	Avascular	2D	2009
[10]	Tumor Growth	Avascular	2D	2010
[11]	Cell-cycle	Avascular	2D	2012
[12]	Tumor Growth	Avascular	3D	2012
[13]	Tumor Growth	Avascular	2D	2013
[14]	Tumor Growth	Vascular	3D	2013
[15]	Tumor Growth	Vascular	3D	2013
[7]	Tumor Growth	Avascular	2D	2014
[16]	Heat Transfer In Tumor	Avascular	2D	2014
[17]	Cancer Growth	Avascular	2D	2014
[18]	Tumor Growth	Vascular	3D	2015

#### a.2. Agent-based modeling

Agent-based modeling (ABM) is another approach to modeling complex systems composed of interacting, autonomous agents. Agents have behaviors, frequently represented by simple rules, and interactions with other agents, which in turn influence their behaviors. Agent-based modeling suggests a technique to model social systems that are composed of agents who learn from their experiences, and adjust their behaviors so they are better suited to their environment [19]. In an agent-based model, each cell is often represented as an agent. Summary of some important published approaches based on ABM is shown in Table 2.

**Table 2:** Summary of some important published agent-based models (M = Migration, P = Proliferation, Q = Quiescence, Mi = Microscopic, Me = Mesoscopic, Ma = Macroscopic).

Ref.	Phenotype	Vascular/Avascular	Dimension	Scale	ODE/PDE	Cancer	Year
[22]	M, P, Q	Avascular	2D	Mi, Me	ODE	Brain	2005
[23]	M, P, Q	Avascular	2D	Mi, Me	ODE	Brain	2006
[24]	A, M, P, Q	Avascular	3D	Mi, Me	ODE	Brain	2007
[25]	A, M, P, Q	Avascular	2D	Mi, Me	ODE	Lung	2007
[26]	A, M, P, Q	Avascular	3D	Mi, Me	ODE	Brain	2009
[27]	M, P, Q	Avascular	2D	Mi, Me	ODE	Brain	2009
[28]	M, P	Avascular	3D	Mi, Me	ODE	Lung	2009
[29]	M, P, Q	Avascular	3D	Mi, Me	ODE	Brain	2011
[30]	A, M, P, Q	Vascular	2D	Mi, Me,Ma	ODE/PDE	Brain	2012
[14]	A, M, P, Q	Vascular	3D	Me, Ma	PDE	Melanoma	2013
[31]	M, H, P, Q	Vascular	3D	Me, Ma	PDE	Breast	2013

ABM is a more realistic modeling approach for many problems, especially problems in which there are multiple types of actors that interact in different ways [5]. However, it can become very intricate when they incorporate a lot of detail. Therefore, it can become computationally expensive, entailing exceedingly long computer run times for simulations and it asks for ancillary developmental resources. Another disadvantage of ABM is that model developers would have to work on the bottom level of abstraction pursue great endeavor to graphical display, memory management and synchronization mechanism [20].

### b. Continuous modeling (mathematical modeling)

Numerous mathematical models of cancer have been developed until the present. Mathematical models play a vital role in the development of knowledge in this field of research, since these models are used to realize the common behavior of a phenomenon in various circumstances, to carry out in silico simulations or experiments, to perform new experiments, and to suggest modifications of theories and test theoretical assumptions [21].

Two primary mathematical methodologies are employed in cancer modeling (ODE and PDE). Partial and ordinary differential equation-based mathematical models of nutrient supply, cancer growth, contaminant and so on, provide a starting point for all mathematical models developed in this study. Ordinary differential equations (ODE) is used to form a description of growth and interactions. ODEs allow the investigator to look at changes in the dynamics of the system in a sense that is matching to how experimental researchers conduct their investigations-that is, the natural production of a system of ODEs is the time course of each interesting variable, just as experimentalists record observations of a system over time. Many of the spatial models in cancer modeling are based on partial differential equations (PDEs) that include tissue stiffness, deformability, spatial heterogeneity and orientational tissue structure. Nonetheless, these methods impose a significant restriction on the time-scales of models. Summary of some important published approaches based on PDE and ODE is shown in **Table 3**.

**Table 3:** Summary of some important published Continuous models.

ODE			PDE		
Ref	Cell Population	Year	Ref	Concentration Modeling of	Year
[32]	Cancer	1964	[33]	Nutrient	2000
[34]	Cancer, Immune	1994	[35]	Nutrient	2001
[36]	Cancer, Immune	2005	[37]	Nutrient	2006
[38]	Cancer, Health	2009	[39]	Oxygen, Glucose	2006
[40]	Cancer, Health	2014	[41]	Oxygen, Glucose	2012

### c. Hybrid modeling

Hybrid modeling is a further extension of the previous studies. The coupled continuum and discrete models in the hybrid modeling framework [1]. The concept of hybrid modeling is discovered on the study of the connection between continuum and discrete modeling expressions. Newly, hybrid modeling techniques have been proposed that combine the advantages of the continuum and the discrete methods to model multiscale problems.

Moreover, hybrid modeling provides more realistic descriptions of microscopic mechanisms while efficiently evolving the entire system to obtain macroscopic observations [42][43][44]. Cancer modeling is one case of such multiscale issue, where the cellular and subcellular scale pathways have been intensively studied and are fairly well understood while the tissue-scale cancer morphology is of interest in clinical applications.

## Proposed model formulation

The following is a description of our model and its sub-models. The sub-models described below simply illustrate how such a hybrid multiscale model can be assembled.

### a. Hybrid multiscale modeling

Cancer evolution is a very complex process, involving many different phenomena, which occurs at different scales. Multiscale models that integrate hierarchies in multiple scales are being established for application in clinical settings [3]. The complexity of cancer development embodies itself at least on three scales: Microscopic, Mesoscopic and Macroscopic (see subsection ‎a.1 to ‎a.3).

Our previous model which was based on an agent, and was published as a paper [2], is highly developed and become more sophisticated in the present paper, and in what follows, a detailed explanation of every aspect of proposed model will be provided.

In our model, cells (agents) are defined in a 3D lattice form (agent space) in which each cell is surrounded by 26 other cells called Moore neighbors.

The single most important defining characteristic of our model agent is its capability to select an action (phenotype) autonomously, that is, to act on its own without external direction in response to situations it encounters (see subsection ‎b).

Substances such as growth factors (VEGF), oxygen, glucose, TGFα and TNFα; and Signaling pathways including EGFR and TNF are described as continuum fields in the cancer microenvironment (see subsection a.2 ‎0and ‎a.1), while individual discrete components (e.g. healthy, cancerous and endothelial cells) dynamically evolve in response to local circumstances like substance concentration.

The spatial scale covers from micrometers to centimeter and the time scale ranges from seconds to hours for microscopic and macroscopic scales respectively.

**Fig 1:**
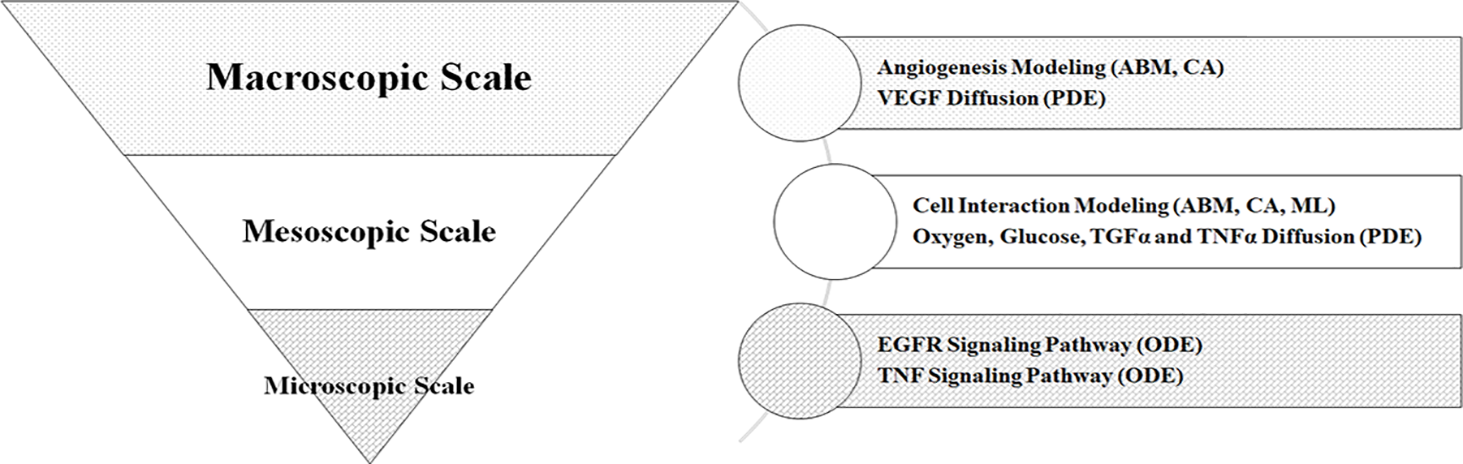
Multiscale model techniques overview. This figure shows the techniques used in each scale (ML = Machine Learning, CA = Cellular Automata, ABM = Agent Based Modeling, PDE = Partial Differential Equation, ODE = Ordinary Differential Equation). Our model combines aspects of both discrete (CA, ABM) and continuum (PDE, ODE) modeling to provide a more complete description of the tumor environment.

**Fig 1** illustrates that three scales, i.e. macroscopic, mesoscopic and microscopic are interconnected because the tumor growth is closely related to cell population density, nutrient concentration, cell behavior and so forth.

#### a.1. Microscopic scale sub-models

The microscopic scale refers to those phenomena that take place at the subcellular level. Signaling pathway is an important element to accede our proposed models to reality because the fate of a cell is determined by signals received from its environment. For this purpose, we used two important signaling pathways (TNF and EGFR) in our model [45][46]. The concentration of each element in the TNF and EGFR signaling are described by ODEs equations. Time step on this scale is second and the concentration of the materials in every point of the grid is updated.

**EGFR signaling pathways:** EGFR Signaling pathway starts with binding TGFα to EGFR and affects cell decision to migrate or proliferate. Each agent evaluates the concentration of EGFR at its current location. In our model, the EGFR signaling pathway of [47] is used and its equations are computed using numerical methods. EGFR Equations related to this signaling pathway are given in S1 Appendix.

The input to EGFR equations is TGFα concentration and their output is PLCγ concentration. After solving signaling pathway equations, the value of PLCγ is calculated. In the case that the PLCγ value of a cell is more than the average of PLCγ concentration of the other cells, it can divide, otherwise it can migrate.

**TNF signaling pathways:** TNF signaling pathway triggers by binding TNFα to TNFR and takes decisions of cell survival and cell death. TNFR is responsible for a diverse range of signaling events within cells, leading to necrosis or apoptosis and inhibiting tumorigenesis. TNF Equations related to this signaling pathway are given in S2 Appendix.

#### a.2. Mesoscopic scale sub-models

The mesoscopic scale refers to the cellular level. The term mesoscopic refers to intermediate between short and long and applies to microstructures to be seen in between the atomic and the macroscopic length scales. In this scale, each cell (or agent) is characterized by a site or position (p(x,y,z)) and phenotype or action. The *p(x*, *y*, *z)* is used to express each site (or point) in the lattice, where *x*, *y* and *z* indicate the integer location in Euclidean terms.

**We have four agents:** healthy cell, cancerous cell, stalk vessel and tip vessel. Transitions between states (or selection a new action) are modeled by an online learning approach (see subsection ‎b). The general idea is to specify such model by means of different states in which it can be, actions which an agent can perform to affect the future behavior, and rewards or costs that depend on the state and decision. After an action has been chosen (based on a learning approach), our model changes its state depending on the action and the current state.

**Cell state (agent state):** The state of each cell is characterized by number of variables (**Table 4**). These variables are based on the simulation environment factors such as the value of signaling pathways output/input (Plcγ, TNFα), nutrients concentration (Oxygen, Glucose, VEGF), number of cell neighbors, etc. The value of each variable is continuous; hence we have an infinite set of states. For each of these variables, we define some rules.

**Table 4:** Variables of state for each agent (for example the variables of the tip vessel state are VEGF, Doubling Age and Number of Capillary Cells).

*Agent* *State Variables*	Healthy Cell	Cancerous Cell	Stalk Vessel	Tip Vessel
**Oxygen Concentration Value**	Yes	Yes	No	No
**Glucose Concentration Value**	Yes	Yes	No	No
**Cell Division Counter**	Yes	No	No	No
**Proliferation Time Delay**	Yes	Yes	No	No
**Hypoxia Counter**	Yes	Yes	No	No
***Plc***_***γ***_ **Value**	Yes	Yes	No	No
**Number of Healthy Moore Neighbors**	Yes	Yes	No	No
**Number of Cancerous Moore Neighbors**	Yes	Yes	No	No
**TNFα Value**	Yes	Yes	No	No
**VEGF Value**	No	No	Yes	Yes
**Tip Cell Age**	No	No	Yes	Yes

**Cell phenotype (agent action):** In the cellular scale, we concentrate on cell behaviors. Each cell (at time t) is assumed to have one phenotype (presented below) according to its type (Cancerous, Healthy, Tip, Stalk). **Table 5** shows the phenotypes of each agent (See [48] for a discussion of the biological theory of this modeling construct).

**Table 5:** Cells can have the following actions based on their types in each site (for example the Tip vessels undergo the actions of branch and expansion).

*Agents* *Actions*	Cancerous Cells	Healthy Cells	Stalk Cells	Tip Cells
**Apoptosis**	No	Yes	No	No
**Hypoxia**	Yes	No	No	No
**Necrosis**	Yes	No	No	No
**Migration**	Yes	Yes	No	No
**Proliferation**	Yes	Yes	No	No
**Quiescence**	Yes	Yes	Yes	Yes
**Branch**	No	No	Yes	Yes
**Expansion**	No	No	No	Yes
**Sprout**	No	No	Yes	No

- **Apoptosis**

Apoptotic healthy cells undergo programmed cell death in response to signaling events [48]. If healthy cells gain apoptotic phenotype, they become inactive and remove from simulation environment.

- **Necrosis**

Necrosis is a form of cancerous cell injury which results in the premature death of cells in living tissue by autolysis. On the contrary, apoptosis is a naturally occurring programmed and targeted cause of cellular death. The only effect of this phenotype will be a grid point occupation, and therefore other cells cannot enter at this point of the grid. By occurring necrosis, necrotic cell not only remains in simulation environment but also preserves from any changes.

- **Migration**

Cell always searches for a place with more nutrition to migrate or to delivers its offspring there. The candidate position (p(x,y,z)) is selected according to the following condition (Eq 1) [14].

$$\vee_{\mathrm{nutrient}}[\mathrm{Max}C_{\mathrm{nutrient}}^{x,y,z}-{\bar{C}}_{\mathrm{nutrient}}^{x,y,z}>3\sigma_{\mathrm{nutrient}}^{x,y,z}]=\mathrm{true}$$ (1)

Where:

∨ is OR operator.
$MaxC_{nutrient}^{x,y,z}$ = maximum Concentration of nutrient among all Moore neighbors of candidate position (p(x,y,z))
${\bar{C}}_{nutrient}^{x,y,z}$ = Average nutrient concentration for candidate position among all its Moore neighbors
$\sigma_{nutrient}^{x,y,z}$ = Standard Deviation of nutrient concentration for candidate position among all its Moore neighbors

$$nutrient=\left\{ \begin{array}{ll} oxygen,\;glucose & For\;Cancerous\;or\;Normal\;Cells\\ VEGF & For\;Vessel\;Cells \end{array}\right.$$

If the candidate site doesn’t have the above condition or two or more candidates have the above condition, all candidate locations will be ranked through Eq 2 and according to their *ProbabilityOfSelection* value, one candidate will be selected randomly.

$${\mathrm{ProbabilityOfSelection}}_{x,y,z}=\frac{{\mathrm{Weight}}_{x,y,z}}{\sum_{\mathrm{for}\;\mathrm{all}\;\mathrm{candidate}\;(x^{\prime },y^{\prime },z^{\prime })}{\mathrm{Weight}}_{x^{\prime },y^{\prime },z^{\prime }}}$$ (2)

Where:

${Weight}_{x,y,z}=\sum_{nutrient}{\bar{C}}_{nutrient}^{x,y,z}$
${\bar{C}}_{nutrient}^{x,y,z}$ = Average nutrient concentration for candidate position among all its Moore neighbors
$nutrient=\left\{ \begin{array}{ll} oxygen,\;glucose & For\;Cancerous\;or\;Normal\;Cells\\ VEGF & For\;Vessel\;Cells \end{array}\right.$

Each lattice site can be occupied by one cancerous/healthy cell or four endothelial (vessel) cells. The cancer cells compete with healthy cells for the empty sites and the cancerous cell outperforms its normal counterpart. Therefore, if a cancerous cell and healthy one simultaneously select an empty site, the cancerous cell will gain the site. If the type of competitors is the same as each other, one of them will be selected randomly. If no movement at all is possible, the cell will remain stationary.

- **Proliferation**

Cell proliferation is the process that results in an increase of the number of cells. Cell proliferation is increased in cancer. Cancerous or healthy cells can enter the proliferation phenotype. In this phenotype, cell divides into two identical offspring cells. In our model, one of the offspring cells is located in their mother grid site and the other one is located in one of the free grid sites in a Moore neighborhood of its mother based on Eqs 1 and 2. However, as soon as its neighboring spaces are occupied by other cells, the cell moves to resting phase (quiescence phenotype) [11,49].

- **Quiescence**

The quiescent phenotype is the default phenotype for a cell. It represents G0 in terms of the cancerous or healthy cells cycle. For endothelial cells, this phenotype means they don’t do anything. It needs to be mentioned here that there is no reverse change of cell from quiescent back to other phenotypes in this model.

- **Hypoxia**

Hypoxia is a phenotype for a cancerous cell in which the cancerous cell is deprived of adequate oxygen supply. Decrease oxygen availability (hypoxia) stimulates cancerous cell to produce VEGF much more. Cancerous cells enter hypoxia when oxygen levels drop below a defined threshold and will enter necrosis if they remain at that level for too long.

- **Branch**

The endothelial cells spearheading the vascular sprouts are known as the endothelial tip cells. Tip cells take the decisions of vessel branch, thereby defining the route in which the new sprout grows [30].

In our model, tip cells deliver two new offspring in two free sites of the grid in its Moore neighborhood of its mother based on Eqs 1 and 2.

- **Expansion**

Tip cells are the leading cells of the sprouts; they guide following endothelial cells (stalk cells) and sense their environment for guidance cues. As the tip cells move out from the existing blood vessel, the stalk cells follow to sprout. As tip cell moves forward, a new stalk cell is created in back of it.

- **Sprout**

Following the tip cells are the endothelial stalk cells, which are highly proliferative, establish adherent and tight junctions to ensure the stability of the new sprout. Stalk cell always searches for a place with more VEGF to deliver its daughter cell there [50]. The candidate place is selected according to Eqs 1 and 2. Type of new vessel generated from the existing stalk cells is the same as its parent. Arteriole and Venule are separated from artery and vein respectively.

**Material diffusion:** We considered five materials in the environment (Oxygen, Glucose, TGFα, VEGF and TNFα). The concentration (C) of each material is identified within each of environment grid points. Environment diffuses the material by PDE diffusion equation based on its source (S), uptake (U) and waste (W). In this model, duty of endothelial cells are the production of Oxygen, Glucose (which is consumed by cancerous/healthy cells) and consumption (or uptake) of VEGF. The duty of cancerous/healthy cells are the production of VEGF (which is consumed by endothelial cells), TGFα and TNFα (which is consumed at mesoscopic scale based on equations in S1 Appendix and S2 Appendix).

- **Oxygen, glucose TGFα and TNFα diffusion**

The concentration of each material is identified within each of environment grid points. Environment diffuses the material by the diffusion equation. We rewrite Diffusion equation for discrete environments in Eq 3 for oxygen, glucose, TGFα and TNFα based on [18] and [51]. For TGFα and TNFα, uptake is computed based on their ODEs equations (see S1 Appendix and S2 Appendix). Grid sites occupied by healthy and cancer cells are sinks of oxygen, glucose, TGFα and TNFα.

$$C_{\mathrm{nutrient}}^{x,y,z}(t+1)=C_{\mathrm{nutrient}}^{x,y,z}(t)+\frac{D_{\mathrm{nutrient}}\times \Delta t}{\Delta S^{2}}\left(\Delta C_{\mathrm{nutrient}}^{x,y,z}(t)\right)-U_{\mathrm{nutrient}}^{x,y,z}(t)+S_{\mathrm{nutrient}}^{x,y,z}(t)$$ (3)

Where:

D_nutrient_ is nutrient diffusion coefficient,
$\Delta C_{nutrient}^{x,y,z}(t)=(\sum_{for\;all\;26\;Moore\;neighbors\;of\;(x,y,z)}C_{nutrient}^{x^{\prime },y^{\prime },z^{\prime }})-{26\times C}_{nutrient}^{x,y,z}$
$U_{nutrient}^{x,y,z}(t)=\left\{ \begin{array}{l} \Delta t\times \zeta_{nutrient}^{x,y,z}(t)\times C_{nutrient}^{x,y,z}(t)\;nutrient=\{oxygen,\;glucose\}\\ 0\;nutrient=\{TNF\alpha ,TGF\alpha \} \end{array}\right.$
$\zeta_{nutrient}^{x,y,z}\left(t\right)=\left\{ \begin{array}{ll} \beta_{c} & cell=cancer,phenotype=proliferation\\ \frac{\beta_{c}}{2} & cell=cancer,phenotype=migration\\ \frac{\beta_{c}}{4} & cell=cancer,phenotype=quiescence\\ \frac{\beta_{c}}{6} & cell=cancer,phenotype=hypoxia\\ \frac{\beta_{c}}{8} & cell=cancer,phenotype=necrosis\\ \alpha_{n} & cell=normal\\ \;0 & \;no\;cell\;exists\;in\;position\;\left(x,y,z\right) \end{array}\right.$
$S_{nutrient}^{x,y,z}(x,t)=\left\{ \begin{array}{l} \left(\frac{\Delta t}{{\Delta S}^{2}}(2\pi \times R^{x,y,z}(t)\times {Pe}_{nutrient}\times (\rho_{nutrient}^{x,y,z}(t)-C_{nutrient}^{x,y,z}(t)))\right)\;nutrient=\{oxygen,\;glucose\}\\ \Delta t\times {SO}_{nutrient}\;nutrient=\{TNF\alpha ,TGF\alpha \} \end{array}\right.$
$R^{x,y,z}(t)=\left\{ \begin{array}{ll} r_{art} & if\;arteriole\;passes\;through\;x,y,z\\ r_{cap} & if\;capillary\;passes\;through\;x,y,z\\ \;0 & no\;vessel\;cell\;passes\;through\;x,y,z \end{array}\right.$
*r*_*art*_ *and r*_*cap*_ *are radius of arteriole and radius of capillary respectively*
$\rho_{nutrient}^{x,y,z}(t)=\left\{ \begin{array}{ll} C_{nutrient}^{art} & if\;arteriole\;passes\;through\;x,y,z\\ C_{nutrient}^{cap} & if\;capillary\;passes\;through\;x,y,z \end{array}\right.$
$C_{nutrient}^{art}\;and\;C_{nutrient}^{cap}\;are\;concentration\;of\;nutrient\;in\;arteriole\;and\;capillary\;respectively$
*nutrient* = {*oxygen*,*glucose*,*TGFα*,*TNFα*}
*β*_*c*_ is constant variable (Table A in S3 Appendix)
Δ***S*** = *the size of grid point*

- **Vascular endothelial growth factor diffusion**

Vascular endothelial growth factor (VEGF) stimulates the ingrowth of a new blood supply from the host vasculature via angiogenesis. Cancerous cells release growth factors (VEGF), in the surrounding normal tissue. We rewrite Diffusion equation for discrete environments in Eq 4 for VEGF.

$$C_{\mathrm{VEGF}}^{x,y,z}(t+1)=C_{\mathrm{VEGF}}^{x,y,z}(t)+\frac{D_{\mathrm{VEGF}}\times \Delta t}{\Delta S^{2}}\left(\Delta C_{\mathrm{VEGF}}^{x,y,z}(t)\right)-U_{\mathrm{VEGF}}^{x,y,z}(t)+S_{\mathrm{VEGF}}^{x,y,z}(t)-W_{\mathrm{VEGF}}^{x,y,z}(t)$$ (4)

Where:

D_VEGF_ is VEGF diffusion coefficient,
$\Delta C_{VEGF}^{x,y,z}(t)=(\sum_{for\;all\;26\;Moore\;neighbors\;of\;(x,y,z)}C_{VEGF}^{x^{\prime },y^{\prime },z^{\prime }})-{26\times C}_{VEGF}^{x,y,z}$
$U_{VEGF}^{x,y,z}(t)=\frac{\Delta t}{{\Delta S}^{2}}\left(2\pi \times R^{x,y,z}(t)\times {Pe}_{VEGF}\times C_{VEGF}^{x,y,z}(t)\right)$
$R^{x,y,z}(t)=\left\{ \begin{array}{cc} r_{art} & if\;arteriole\;passes\;through\;x,y,z\\ r_{cap} & if\;capillary\;passes\;through\;x,y,z\\ 0 & no\;vessel\;cell\;passes\;through\;x,y,z \end{array}\right.$
*r*_*art*_ *and r*_*cap*_ *are radius of arteriole and radius of capillary respectively*
$S_{VEGF}^{x,y,z}(x,t)=\Delta t\times \zeta_{VEGF}^{x,y,z}(t)$
$\zeta_{nutrient}^{x,y,z}\left(t\right)=\{\begin{array}{ll} \phi_{c} & cell=cancer,phenotype=necrosis\\ \frac{\phi_{c}}{2} & cell=cancer,phenotype=hypoxia\\ \frac{\phi_{c}}{4} & cell=cancer,phenotype=quiescence\\ \frac{\phi_{c}}{6} & cell=cancer,phenotype=migration\\ \frac{\phi_{c}}{8} & cell=cancer,phenotype=proliferation\\ \;0 & no\;cancerous\;cell\;exists\;in\;position\;\left(x,y,x\right) \end{array}$
$W_{VEGF}^{x,y,z}(t)=\Delta t\times \omega_{VEGF}\times C_{VEGF}^{x,y,z}(t)$
*ω*_*VEGF*_ and *ϕ*_*c*_ are constant variables (Table A in S3 Appendix)
Δ***S*** = *the size of grid point*

#### a.3. Macroscopic scale sub-models

The macroscopic scale pertains to processes happening at the tissue level. At the tissue level, we consider vessels and tumor directly.

**Fig 2:**
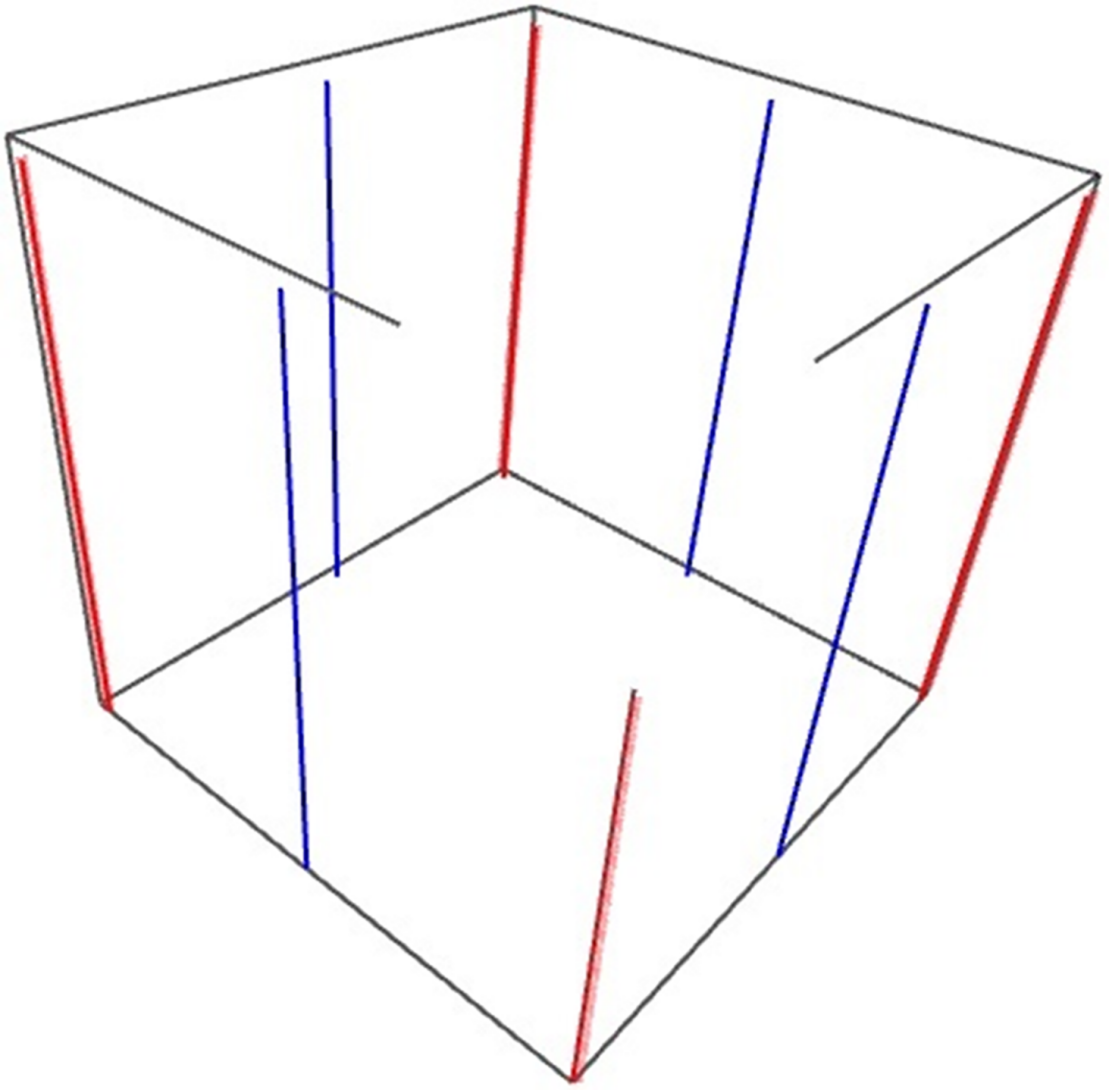
Four veins and four arteries located on the middle of four surfaces and four edges of cube.

**Angiogenesis:** Angiogenesis is termed as the growth of new blood capillaries on the basis of pre-existing vessels, which is a critical step in cancer growth and metastasis. Tumorigenesis is a prime case of an emergent tissue patterning phenomenon reliant on many cell types, both normal and aberrant cell behaviors. Models of angiogenesis fall into three classes: models of vasculature growth without tumor or other tissue cells, models of vasculature growth with only tumor cells, and models of vasculature growth with both tumor and healthy tissue cells [31]. Vasculatures in the system give cells (agents) materials (oxygen, glucose, TGFα and TNFα), which they require to survive. Once vascularized, cancer has access to a huge nutrient source and rapid growth ensues. At the other hand, VEGF stimulates the ingrowth of a new blood supply from the host vasculature via angiogenesis. In this section, we present a model of angiogenesis and vascular tumor growth. We assume that there are eight main parallel parent vessels (four veins and four arteries located on the four vertical edges and four vertical surfaces of cube respectively) as the major part of the blood supply in our 3D simulation environment as shown in **Fig 2**.

In our model, the general order of blood flow is as follow: Arteries → arterioles → venules → veins. When we refer to an “active” vessel, we are referring to an arteriole in which it can connect to a venule. As arteriole connects to venule, the blood flow circulated throughout it.

### b. Q-Learning based on SVR-NSGA-II

There is no information available in the literature of the quantitative relationship between the phenotype rate and nutrient density and time. So we need a practical computational method for constructing autonomous systems that improve themselves with experience.

Markov decision processes (MDPs) provide a mathematical framework for modeling decision making in situations where results are partly random and partly under the control of a decision maker. Reinforcement learning (RL) can solve Markov decision processes without explicit specification of the transition probabilities. RL is an area of machine learning inspired by behaviorist psychology, concerned with how software agents ought to determine the ideal behavior within a specific environment, in order to maximize its performance. A RL agent interacts with its environment in discrete time steps.

There are many different algorithms that tackle this issue. Q-learning is one type of algorithm used to calculate state-action values. It estimates a function which measures the goodness of all possible actions, and use that function to define the policy.

**Fig 3:**
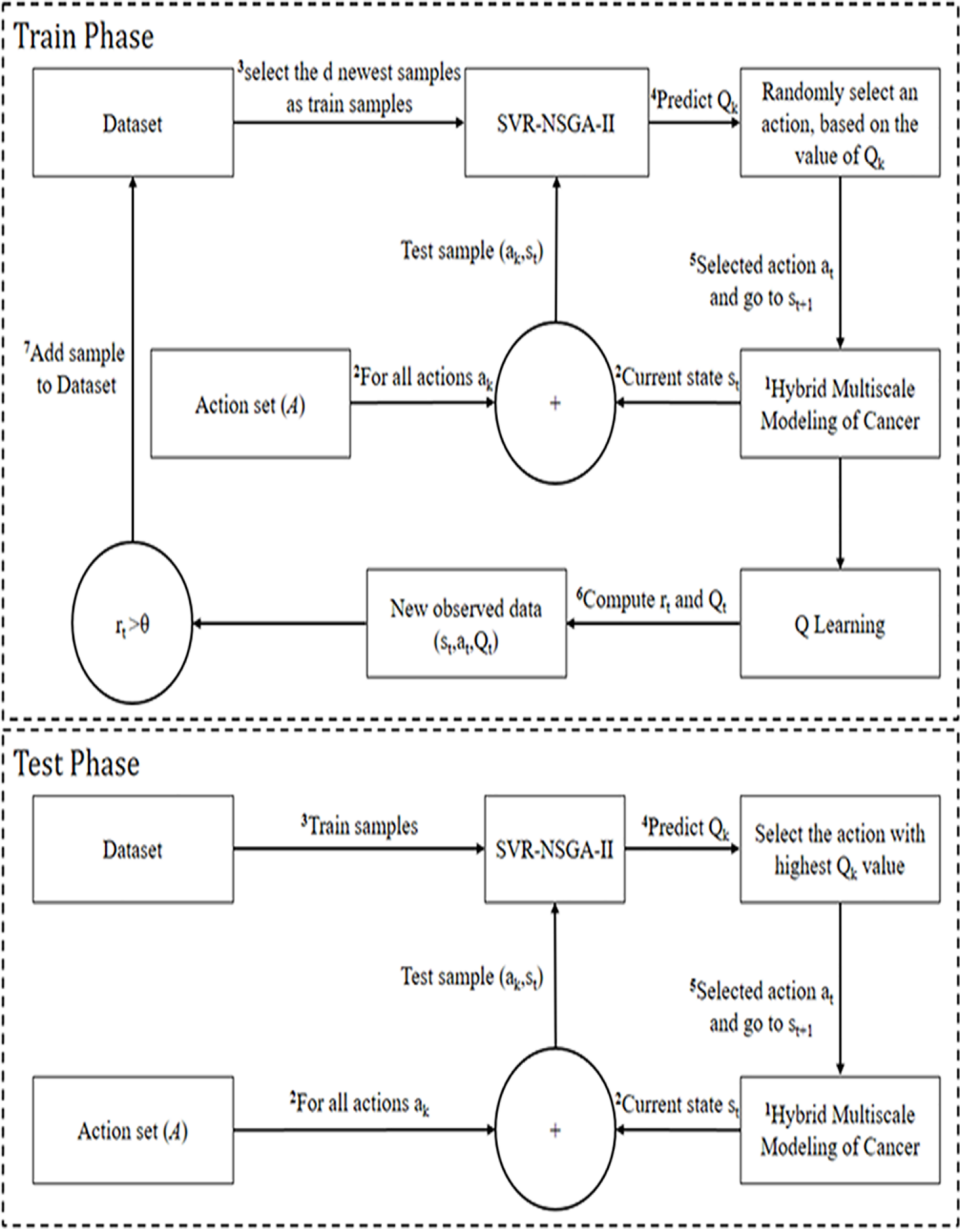
Sketch map of Q-learning based on SVR-NSGA-II. In train phase, a simulated dataset is generated which is used by SVR-NSGA-II to determine cancer cell behaviors autonomously. Model performance assessment is done through test phase.

Discretization and value function approximation are the commonly methods to solve continuous space problems in reinforcement learning. We use SVR-NSGA-II to approximate the Q value of state-action pair. In other words, since the variables of cell states are continuous (**Table 4**) then the state space is continuous and to solve a continuous-state space, we use SVR-NSGA-II. The model used in this work is summarized in **Fig 3**.

#### b.1. Regression sub-model (SVR-NSGA-II)

**I**t is well known that SVR generalization performance (estimation accuracy) depends on a good setting of *C*, Ԑ and kernel function parameter (e.g. gamma). Existing software implementations of SVM regression usually treat these parameters as user-defined inputs. Selecting a particular kernel type and kernel function parameters are usually based on application-domain knowledge and also should reflect the distribution of input (x) values of the training data.

Parameter C decides the tradeoff between the model complexity (flatness) and the degree to which deviations larger than Ԑ are tolerated in optimization formulation. Parameter Ԑ controls the width of the Ԑ-insensitive zone, used to fit the training data. The value of Ԑ can affect the number of support vectors used to construct the regression function. Parameter gamma (γ) is the free parameter of some kernels (e.g. RBF, polynomial and sigmoid kernels).

Kernel function maps the data into higher dimensional spaces in the hope that in this higher-dimensional space, the data could become more easily separated. Each kernel function can extract a specific type of information from a given dataset. Now the question arises how to get an appropriate kernel function. Since each kernel has some degree of variability in practice, there is nothing else for it but to experiment with different kernels and regulate their parameters.

In a previous study, we proposed a method (SVR-NSGA-II) [52] that is an improved algorithm based on SVM. SVR-NSGA-II optimizes the above parameters (C, Ԑ and γ) by implementing the evolutionary process (NSGA-II). The Non-dominated Sorting Genetic Algorithm II (NSGA-II) is a Multiple Objective Optimization (MOO) algorithm and is an instance of an Evolutionary Algorithm from the field of Evolutionary Computation.

For single objective optimization problems, the best single design is the goal. But for multi-objective problems, with several and possibly conflicting objectives, there is usually no single optimal solution. A suitable solution should provide for acceptable performance over all objectives.

We convert the RL problem into regression problem, which the observed states and actions are viewed as input variables and the Q values as output variables. To collect enough training samples, simulation iterations must be high enough for accurate results. In order to improve learning performance, the training samples are slide with the manner of time-window and the newest samples are considered as train dataset.

Assume that we are given a *training set* D, consisting of pairs *(x*_*i*_,*y*_*i*_*)*, for i = 1,…,m. Each *sample x*_*i*_ is a vector that describes the cell states, phenotypes (action) and Q values. In other words, the phenotypes, the variables of cell states, constitute the features of the dataset. The label *y*_*i*_ associated with *x*_*i*_ is a discrete value between -1 and 1 and describe the Q value.

An initial population of chromosomes is generated randomly. Then the population is evolved for a number of generations. The chromosome is a fixed length list of genes or parameters.

We have implemented three kernels Sigmoid, Polynomial and RBF for our method. SVR-NSGA-II aggregate these three kernels which are performed through a simple voting based on allocating weight to each of them. This weight is based on F-Measure of its kernel for train dataset (Eq 5).

F-measure is a harmonic mean between precision (or Confidence) and recall (or Sensitivity). Recall is the percentage of real positive cases that are correctly predicted positive. Precision indicates the percentage of predicted positive cases that are correctly real positives. Also, NSGA-II optimizes Recall, F-Measure and precision in a multi-objective way.

$${\mathrm{Weight}}_{k}=F-\mathrm{Measure}=\frac{2\times \mathrm{Recall}\times \mathrm{Precision}}{\mathrm{Recall}+\mathrm{Precision}}$$ (5)

where:

$Recall\;(or\;Sensitivity)=\frac{TP}{TP+FN},\;Precision\;(or\;Confidence)=\frac{TP}{TP+FP}$
*TP is the number of correct predictions in which an instance is positive*;
FN is the number of incorrect predictions in which an instance is negative
FP is the number of incorrect predictions in which an instance is positive
TN is the number of correct predictions in which an instance is negative
*k =* {*Sigmoid*,*RBF*,*Polynomial*}

#### b.2. Q-learning sub-model

Q-learning is a simple incremental algorithm developed from the theory of dynamic programming for delayed reinforcement learning. The objective of Q-learning is to quantify the Q value for an optimal value when the system dynamics features are unexplored. Q-learning approach is fulfilled as follows: a Q-learning system observes the current state *s*_*t*_ and executes an action *a*_*t*_ at each time step *t*. Next, perceive the subsequent state *s*_*t+1*_ and receives an immediate reward *r*_*t*_. The value of Q can be adapted based on Eq 6 where η is a learning rate that controls the learning speed and γ is a discount factor used to determine the proportion of delay to the future rewards.

$$Q(s_{t},a_{t})\leftarrow (1-\eta )Q(s_{t},a_{t})+\eta [r_{t}+\gamma (\max_{a_{t+1}}\left(Q(s_{t+1},a_{t+1})\right))]$$ (6)

**Policy (cellular phenotype decision):** To enable healthy/cancerous cells to evolve in the 3D domain, we need to define some rules. These rules clarify the relationship between cell states (see **Table 4**) and cell actions (see **Table 5**). For each rule, if the probability of selection for some phenotypes increases, it decreases for other phenotypes. In **Table 6**, policies are determined on a scientific basis using evidences derived from various studies.

**Table 6:** *The relationship between state variables and actions according to policies*.

No.	Description	Ref.
1	Elements occupied by cancerous/healthy cells whose local **oxygen** concentration falls below threshold are more likely to select **hypoxia**, **necrosis** (for cancerous cell) or **apoptosis** (for healthy cell) phenotypes; On the contrary, if the oxygen level goes above threshold, the selection of these phenotypes will reduce and choosing proliferation, as well as migration, will increase.	[53]
2	**Glucose** has two thresholds (dead threshold and active threshold). If the concentration of glucose for cancerous/healthy cell at its current site, is greater than the active threshold, the agent will pick **proliferation** and **migration** with more probability than other actions. If the concentration of glucose is less than the dead threshold, the agent has more chance to select **necrosis**/**apoptosis** phenotype. If the concentration is between active and dead thresholds, the healthy cell will enter into **quiescence** with more probability.	[30]
3	Since healthy cells had a limited number of divisions (**cell division counter**), when number of cell division goes above the cell division threshold, quiescence selection probability for healthy cell rises.	[54]
4	As cancerous/healthy cells select proliferation phenotype, they need minimal amounts of oxygen. If a sufficient amount of oxygen is not available, cell postpones the proliferation and as soon as the conditions are met, it can proliferate. By increasing the **proliferation time delay**, the probability of **hypoxia**, **apoptosis**/**necrosis** increases too.	[55]
5	If the cells lacked oxygen for a period of time greater than the hypoxia threshold, the cancerous/healthy cell has more chance to select **necrosis**/**apoptosis** phenotype. If this time is less than hypoxia threshold and greater than 1, the probability of choosing **hypoxia** phenotype increases. In hypoxia phenotype, the healthy cell has a little chance to survive.	[50]
6	If the value of **Plcγ** inside a site occupied by an agent is less than the average value of Plcγ of all other sites, the agent will start to **migration** with more chances. Otherwise, the probability of **proliferation** is more feasible than the other phenotypes.	[30]
7	The more external healthy/cancerous Moore neighbors a cell has, the more likely it is to migrate. As the **number of healthy/cancerous Moore neighbors** increases, the probability of **migration** increases too. In the study, only if the cell has more than two neighbor cells (threshold = 2), it will migrate with a positive chance.	[55]
8	As **TNFα** concentration increases, the probability of cell survival decreases and the probability of cell death increases. Therefore, by increasing TNFα concentration higher than a threshold, the probability of choosing **necrosis** or **apoptosis** increases.	[56]
9	The concentration of **VEGF** plays a direct role in a **sprout** of stalk cells. If levels of VEGF higher than a threshold, it can lead to increased sprout.	[57]
10	New tip sprouts must mature for a length of time (**tip cell age**) at least equal to ψ before being able to branch. Having reached this age, the probability of **branching** for tip sprouts increases. On the contrary, the probability of **expansion** and **quiescence** for tip sprouts decreases.	[58]

**Fig 4:**
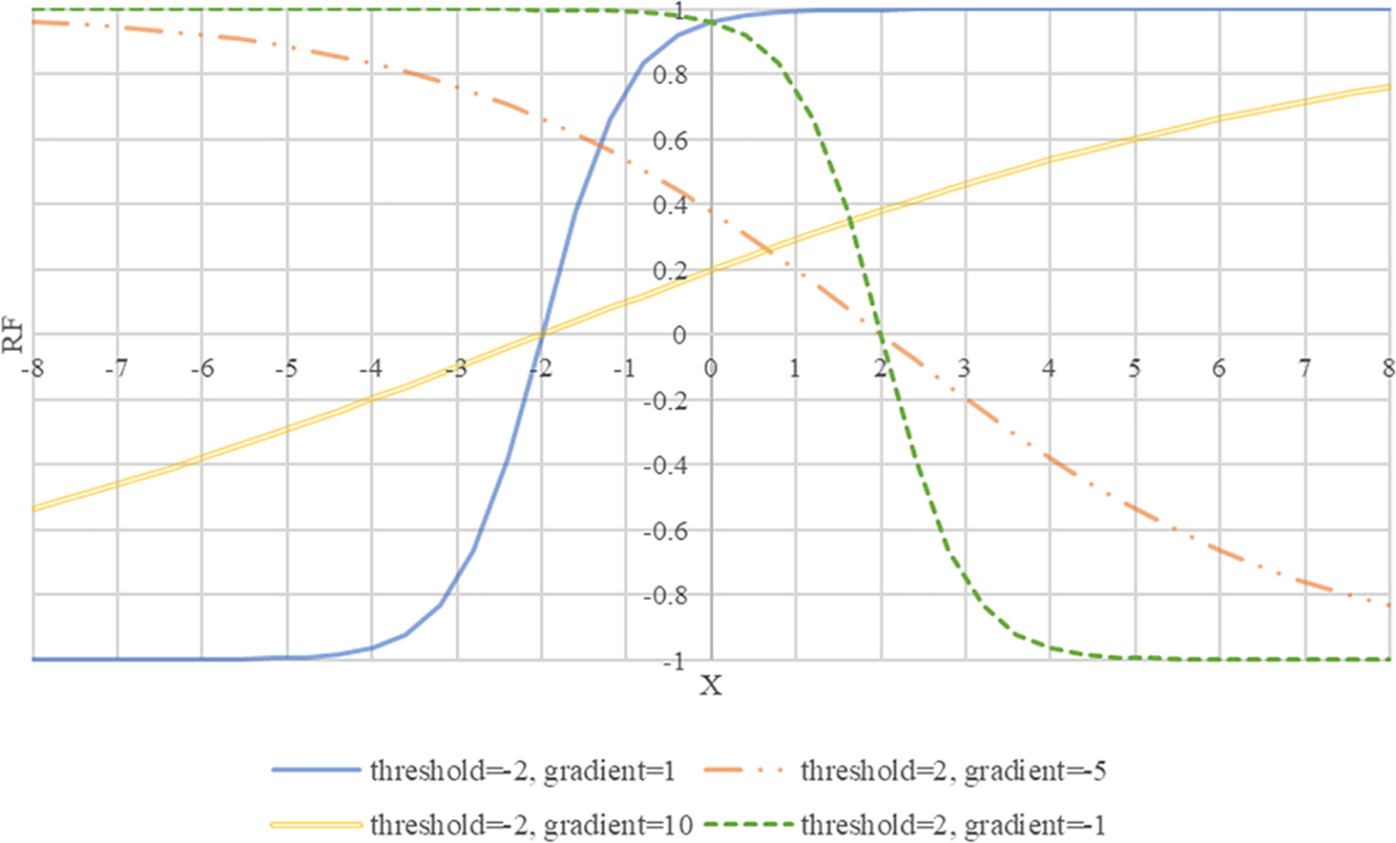
Four reward functions examples. Threshold point (or inflection point) is a point on a curve at which the curve changes from being concave (concave downward) to convex (concave upward), or vice versa. Gradient (or slope) specifies the rate of RF change.

**Reward:** The objective of the learner is to choose actions maximizing discounted cumulative rewards over time. The value of reward at time t (r_t_) is calculated based on the following reward function (RF). In Eq 7, for each policy (p) the value of the reward function is equal to 0 when the value of x is equal to the threshold and also its sign changes at this point. It is obvious that the value of RF is between -1 and +1. A gradient is a constant number that describes both the direction and the steepness of the line. RF is increasing/decreasing if it goes up/down from left to right if the gradient is positive/negative respectively (see **Fig 4**).

$${RF}_{p}(x)=tanh\left(\frac{x-{threshold}_{p}}{{gradient}_{p}}\right)$$ (7)

where:

x equal to value of state variables

In Table B in S3 Appendix, we specify a threshold for all rules that the sign of its probability changes at this point. We use the average of above function for all policies to calculate the total value of the reward.

### c. Algorithm steps

Our model has two phases (train and test). During train phase, the dataset is built and finally, our model is run in the test phase to get the results. The Q-learning based on SVR-NSGA-II can be generalized as follows (see **Fig 3**):

#### Train phase

*Step 1*. *Check the current state s*_*t*_
*Step 2*. *Construct test sample (s*_*t*_,*a*_*k*_*) comprised of each action a*_*k*_ *in action set (A) and current state s*_*t*_
*Step 3*. *Constitute dataset for training (select the d newest samples)*
*Step 4*. *For all available k actions*, *predict Q*_*k*_ *corresponding to (s*_*t*_,*a*_*k*_*) by SVR-NSGA-II*
*Step 5*. *Randomly select action a*_*t*_ *based on its Q*_*t*_ *value (*$\mathrm{Prob}(a_{t})=\frac{Q_{t}}{\sum_{i=1}^{\left|A\right|}Q_{i}}\;where\;a_{t}\in A$*)*
*Step 6*. *Run proposed hybrid multiscale model (run diffusion and signaling pathways) and perform action a*_*t*_, *obtain r*_*t*_ *and the successor state s*_*t+1*_
*Step 7*. *Update Q*_*t*_ *according to Eq 6*
*Step 7*. *Add the new observed data into dataset (s*_*t*_, *a*_*t*_, *Q*_*t*_*) if r*_*t*_*>θ (θ is a predefined threshold value)*

#### Test phase

*Step 1*. *Check the current state s*_*t*_
*Step 2*. *Construct test sample (s*_*t*_,*a*_*k*_*) comprised of each action a*_*k*_ *in action set (A) and current state s*_*t*_
*Step 3*. *Constitute dataset for training*
*Step 4*. *For all available k actions*, *predict Q*_*k*_ *corresponding to (s*_*t*_, *a*_*k*_*) by SVR-NSGA-II*
*Step 5*. *Select action with highest Q*_*t*_ *value*
*Step 6*. *Run proposed hybrid multiscale model (run diffusion and signaling pathways) and perform action*

## Analytic results

In the results presented here, the simulation starts from the initial tumor and microvascular network and finishes at 21 days when the tumor size reaches finishing criteria. Vasculatures added in the lattice provide a continuous source of the nutrient.

### a. Lattice setup

We simulate our model in a comparable size of lattice and show that the findings are in good agreement with biological tumor behavior. We choose a cubic geometry for the 3D tumor lattice and the Moore neighborhood. Each agent represents a single cell and exists on a three-dimensional grid to approximate a tissue. The lattice spacing is 20 μm, which is approximately the diameter of cells. The 3D virtual tumor environment is made up of a discrete lattice consisting of a grid of 40 × 40 × 40 points.

### b. Model initialization

To begin with, in a circle with radius 60μm at the center of the lattice, cancer cells are initialized with a probability of 70 percent. Initial proportions of normal cells in the lattice are obtained with a probability of 70 percent. Also, oxygen and glucose are normally distributed with high density near eight preexisting vessels. We have estimated our model parameters through a deep search of the theoretical and experimental biology and clinical literature [59–61]. We summarize those estimates in tables within S3 Appendix.

### c. Simulation results

Computational simulations of the model were performed in order to analyze its performance. Model execution is based on an iterative process, with each tick or step iteration representing approximately one hour in real time. The output data are the time series of the density and amount of each type of cell.

**Fig 5:**
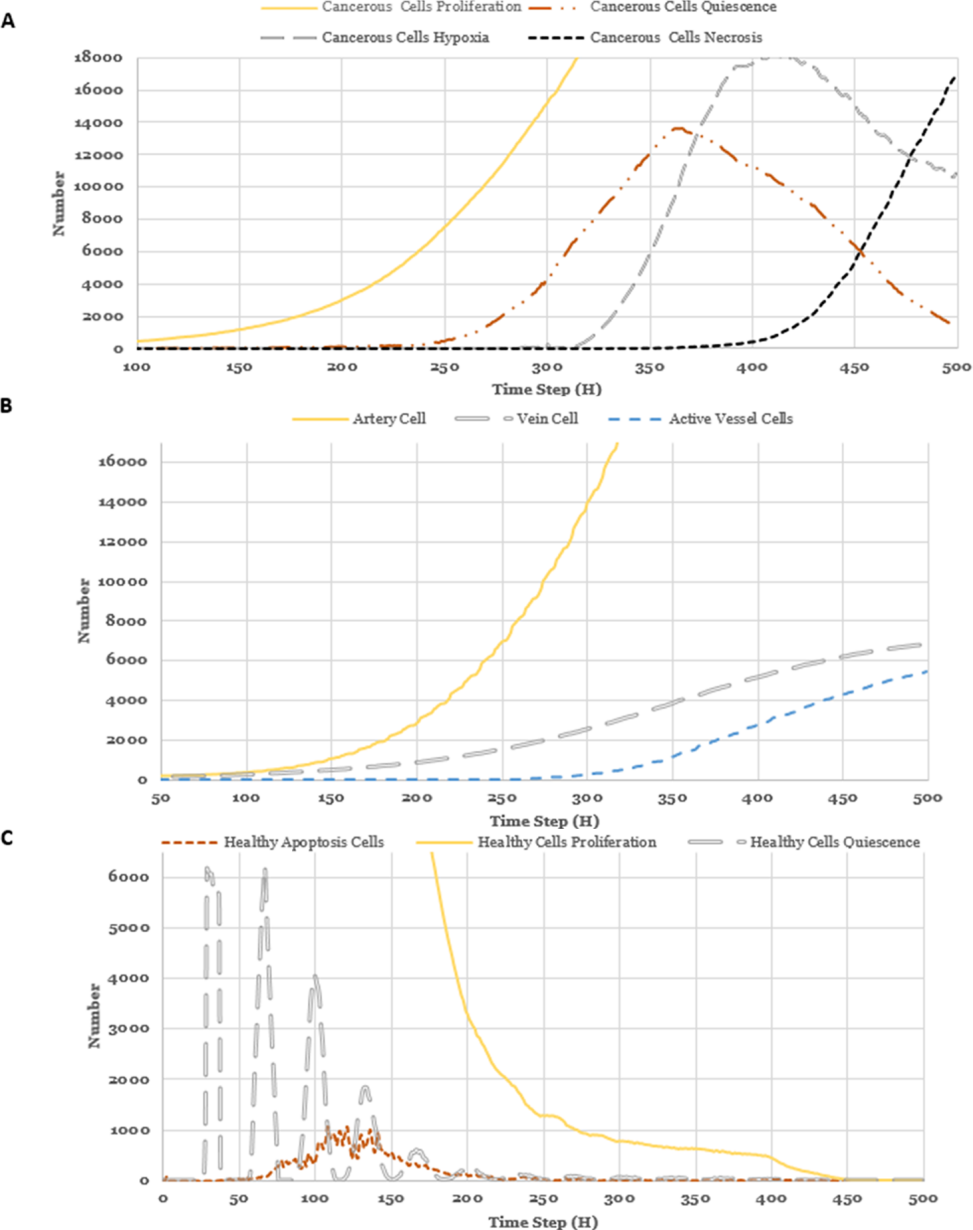
Evolution of Cancerous cells actions (A), number of vessel cells (B) and healthy cells actions (C).

Prolonged hypoxia of the tumor tissue leads to necrosis, and necrotic regions are also characteristic of solid tumors [62]. Tumor necrosis is a sign that rapid tumor cell proliferation continues and cancerous proliferation occurs at a much higher speed compared to cancerous necrosis [63] (**Fig 5A**). Initially, due to insufficient vascularization, most of the tumor cells are quiescent and secrete VEGF which stimulates an angiogenic response(**Fig 5B**). As the tumor cells grow, some of the cells within the tumor mass will be starved of oxygen and eventually become hypoxic. Initially, insufficient nutrient supply in areas at distance from the vessels causes the prevalent death of the healthy cells (**Fig 5C**).

**Fig 6:**
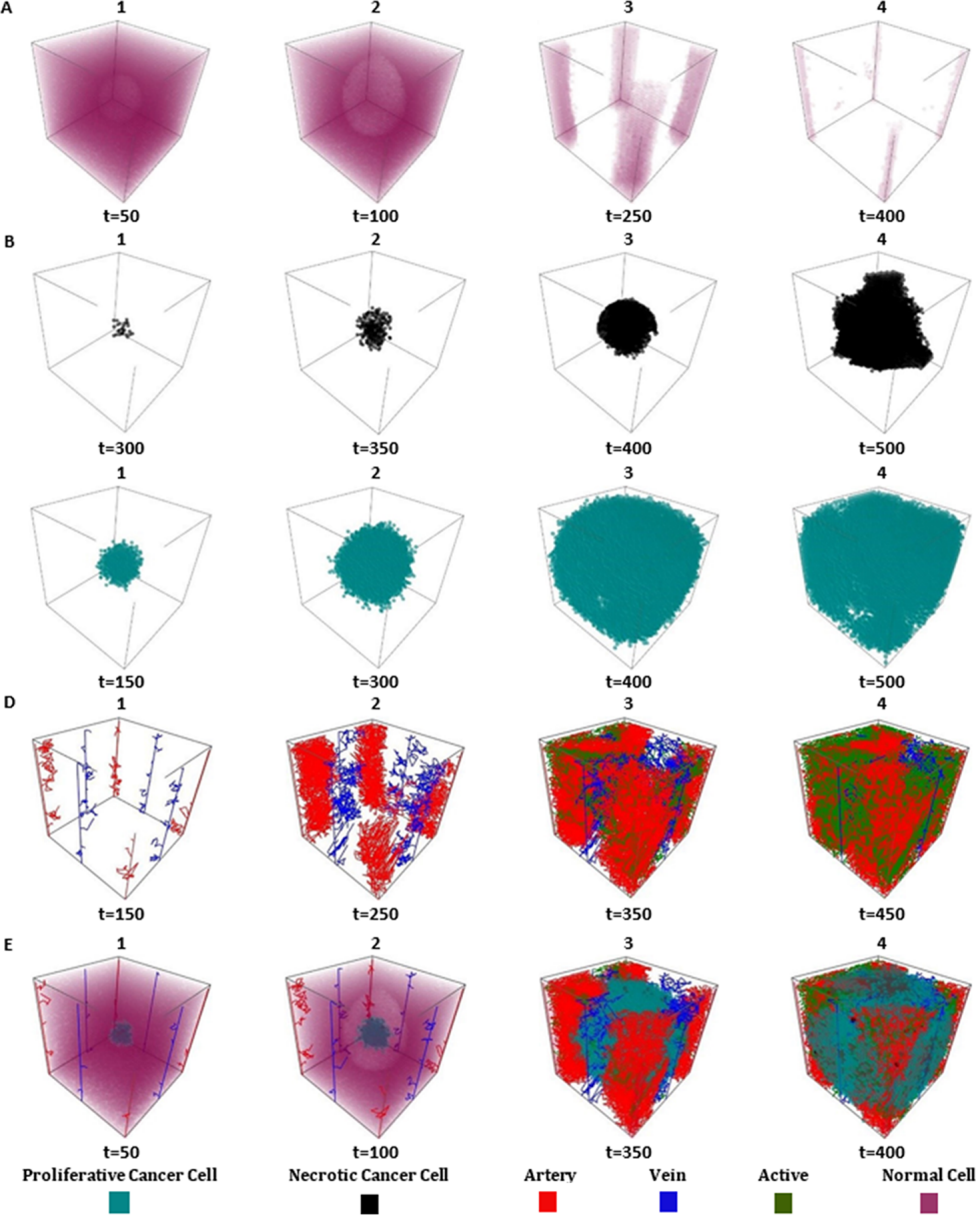
Growth pattern of cells. A: series of images showing the growth pattern of normal cells after 50, 100, 250 and 400 time steps. B: snapshots of Spatio-temporal evolution of necrotic cancer cells. C: snapshots of Spatio-temporal evolution of proliferative cancer cells. D: 3D vasculature of vessels. The vessels labeled in red and blue are arteries and veins respectively. E: Graphical visualization of tumor morphologies at different time with a 3D model.

Spatial distribution of the healthy cells at different times are shown in **Fig 6A**. The normal cells that surround the tumor are faded out gradually. From **Fig 6B**, it can be seen that the necrotic cells are scattered all around the necrotic center of a tumor as it is usually observed in clinical biopsies. The time-spatial patterns reveal a tumor with a compact shape and irregular boundaries, as occurs in some solid tumors [9]. Cells at the center of tumor suffocate because of lack of oxygen and die (necrosis), forming a necrotic core.

Additional proliferation (see **Fig 6C**) leads to its vascular stage, where cancer cells enhance the existing vascular network through angiogenesis. The outcomes from the simulation, showing the development of a tumor and its associated network of blood vessels, are depicted in **Fig 6D**. Vessels emerge near the initial tumor and form a well-vascularized tumor; far from the four initial artery vessels, most normal cells have died.

At the beginning, cancerous cells spread further than healthy cells. However, in the end, there were still more cancer cells than normal cells. **Fig 6E** shows how the number and spatial distribution of the different cell types evolve over time. A colony of cancer is free to grow isotopically until it occupies all the space available. Cancerous cells eventually spread over the whole tissue and killing almost all of the normal cells. More figures are presented in S4 Appendix.

## External validation

Model accuracy can only be established through external validation. A number of ODE models have been proposed to represent tumor growth [64][65]. Gompertzian growth has been one of the most studied decelerating tumor growth over the past 60 years [66]. It is a type of mathematical model for a time series, where growth is slowest at the start and end of a time period (Eq 8). The predictive performance of the Gompertz model has been validated by many researchers [67]. An important validation of our model is shown by comparing the Gompertzian α parameter of simulated tumors with the corresponding parameter of actual tumors. The author in [68] proves that the value of α and β must be in the range of [0.005, 0.016] and [0.121, 0.390] respectively.

$$n(t)=n_{0}\left(e^{\frac{\alpha }{\beta }(1-e^{-\mathrm{\beta t}})}\right)$$ (8)

*Where:*

n(t) is cancerous cell population at time t, n_0_ is cancerous cell population at t = 0, α is a parameter that corresponds to instantons growth rate of n(0) and β is a parameter that measures how rapidly curve departs from a simple exponential curve into the sigmoid shape.

In [63] a function depicting vessel growth over time was created from experimental data to define growth within the computational model (Eq 9). This function depicts the total vascular length within the domain at any point in time. A sigmoid curve was chosen to characterize vessel growth as such a curve is often used to describe population growth restricted by limited resources.

$$g(t)=g_{0}+\frac{\alpha }{1+e^{-\frac{t-t_{1/2}}{\beta }}}$$ (9)

*Where:*

g(t) is the total vascular length at time t, g_0_ is the initial vessel length at t = 0 (bottom of the sigmoid curve), α is the range of the function (top minus bottom) and β is the slope of the curve. t_1/2_ is the time at which g(t) is halfway in between the top and bottom of the sigmoid curve.

Our model also requires a function describing vessel branching over time. In [63] an exponential function was used to describe branch formation as branching metric data taken during the 7 day culture period (Eq 10).

$$b(t)=b_{0}+\alpha e^{\mathrm{\beta t}}$$ (10)

*Where:*

b(t) is the number of branch points at time t, b_0_ is the initial number of branches at t = 0, α scales the exponential term and β describes the rate of branch formation. We consider the value of α and β equals to 2.62 and 0.105 respectively, where these parameters are determined experimentally [59].

**Fig 7:**
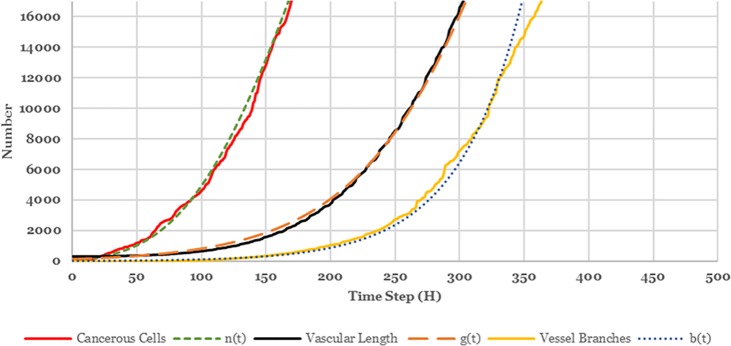
External validation of our model. Parameters were chosen as follows: ((n_0_, α, β) = (100,0.01,0.3), (g_0_, α, β,t_1/2_) = (0,58500,62,360), (b_0_, α, β) = (0,2.62,0.105)).

**Fig 7.** shows that the tumor growth, vessel growth and vessel branching obey these three functions. It shows cancer cells grow exponentially in the beginning. As soon as all empty sites in lattice are occupied by cancer cells, migration or proliferation phenotypes cannot be selected anymore (see subsection ‎a.2). Since the grid in our simulation has 64000 sites, it has enough space for first 504 hours (21 days) time step. After 504 hours time step, there isn’t any empty site for cancerous cells to grow and exponential growth converts to exponential decay. Finally, the lack of space leads to cessation of cancer cell growth.

In order to assess the learning performance of the proposed model, we must evaluate our model at the microscopic level. The Root Mean Square Error (RMSE), Mean Absolute Deviation (MAD) and Mean Absolute Percentage Error (MAPE) are used to compare the fits of different forecasting and smoothing methods. RMSE is a frequently used measure of the difference between values predicted by a model and the actual values (i.e. n(t), b(t) and g(t)). RMSE more aggressively punishes big errors than small ones. MAD expresses accuracy in the same units as the data, which helps conceptualize the amount of error. MAPE is useful to compare the precision between different methods.

This work compares the proposed algorithm with other approaches. RMSE, MAD and MAPE of our proposed method and three other methods are given in **Table 7**. In this table, it is obvious that all the three error measures are significantly improved through utilizing kernel aggregation instead of the single kernel. The results of this study suggest that our proposed model can outperform a human observer in recognizing cancer.

**Table 7:** The quantitative results for proposed classification of the cell (SN = SVR-NSGA-II).

		Q-Learning System Based on						
		SN	SN-RBF	SN-Poly	SN-Sigmoid	Simple SVM	MLP	C4.5
n(t)	**RMSE**	414.64	604.64	938.64	738.64	793.51	961.25	1618.96
	**MAD**	3567.59	4288.59	5976.59	5203.59	7833.60	9496.96	16003.09
	**MAPE (%)**	25.16%	29.16%	38.16%	34.16%	36.02%	43.70%	76.82%
b(t)	**RMSE**	49375.11	51149.11	56033.11	53582.11	62821.42	64152.38	66366.72
	**MAD**	15951.88	17127.88	20210.88	18748.88	24019.75	24968.11	25791.15
	**MAPE (%)**	28.48%	30.48%	34.48%	32.48%	36.86%	38.61%	39.53%
g(t)	**RMSE**	252.77	306.77	353.77	325.77	1705.05	1883.63	2384.43
	**MAD**	208.41	225.41	294.41	247.41	1128.49	1241.26	1689.78
	**MAPE (%)**	8.99%	9.37%	10.62%	9.46%	11.43%	11.79%	15.54%

## Conclusive remarks and future directions

We have developed a new computational modeling paradigm for predicting the behavior resulted from the interaction of cells. Our proposed approach is expected to be a cancer modeling and simulation framework. We incorporate the composition of cells, intercellular and intracellular adhesion as well as processes involved in cell cycle (quiescence, proliferation, hypoxia, necrosis, apoptosis, and migration) in our model, which can accurately simulate angiogenesis.

It has the method of representing biological cells as autonomous software agents. The most important features of our model are the capability of cells to select their phenotype intellectually. We propose an automatic cell phenotype prediction procedure in the study presented here, that predicts the value of Q by using Q-learning and SVR-NSGA-II methods.

A new Q-learning method is proposed, whose rules capture some generic features of tumor development, to study the influence of environmental conditions on the evolution of a tissue containing healthy and cancerous cells. A simulated dataset, which includes the information of cell phenotypes, is generated from the simulation environment (see S5 Appendix). Finally, we attack the classification problem (phenotype selection) using the regression approach, which is done by SVR-NSGA-II.

The model proposed in this study is capable of capturing the Gompertzian behavior of tumor growth. In addition, measurements of vessel growth and branching provided by simulation has excellent statistical agreement with experimental data.

There are some limitations of the current model. Firstly, the neighboring cells of cancer cells behave differently to the same cells in a normal context, but it can play an active role in controlling the behavior of cancer cells. Secondly, this model doesn’t deal with changes in tissue architecture and represents tissue structure on a fixed grid in order to simplify the calculation. The dynamic changes in tissue topology in the presence of competing cells would be followed in our next paper. Further model extensions could include incorporating a more detailed description of subcellular level and tumor-induced angiogenesis.

## Supporting information

S1 Appendix (DOCX)

S2 Appendix (DOCX)

S3 Appendix (DOCX)

S4 Appendix (DOCX)

S5 Appendix (RAR)
