# Supplementary material for: Hybrid multiscale modeling and prediction of cancer cell behavior
Source: PLoS One. 2017 Aug 28;12(8):e0183810. doi: 10.1371/journal.pone.0183810 (PMC5573302; doi:10.1371/journal.pone.0183810)
Supplement: S1 Appendix — (DOCX) [file pone.0183810.s001.docx]

# S1 Appendix

**EGFR signaling pathway**: Kinetic equations comprising the computational model for EGFR signaling pathway [47].

$$\frac{d\left[ EGF \right]}{dt}= -v_{1}$$

$$\frac{d\left[ R \right]}{dt}= -v_{1}$$

$$\frac{d\left[ Ra \right]}{dt}= v_{1}- 2v_{2}$$

$$\frac{d\left[ R2 \right]}{dt}= v_{2}+ v_{4}- v_{3}$$

$$\frac{d\left[ RP \right]}{dt}= v_{3}+ v_{7}+ v_{11}+ v_{15}+ v_{18}+ v_{20}- v_{4}- v_{5}- v_{9}- v_{13}$$

$$\frac{d\left[ R-PL \right]}{dt}= v_{5}- v_{6}$$

$$\frac{d\left[ R-PLP \right]}{dt}= v_{6}- v_{7}$$

$$\frac{d\left[ R-G \right]}{dt}= v_{9}- v_{10}$$

$$\frac{d\left[ R-G-S \right]}{dt}= v_{10}- v_{11}$$

$$\frac{d\left[ R-Sh \right]}{dt}= v_{13}- v_{14}$$

$$\frac{d\left[ R-ShP \right]}{dt}= v_{14}- v_{24}- v_{15}- v_{17}$$

$$\frac{d\left[ R-Sh-G \right]}{dt}= v_{17}- v_{18}- v_{19}$$

$$\frac{d\left[ R-Sh-G-S \right]}{dt}= v_{19}- v_{20}+ v_{24}$$

$$\frac{d\left[ G-S \right]}{dt}= v_{11}+ v_{23}- v_{12}- v_{24}$$

$$\frac{d\left[ ShP \right]}{dt}= v_{15}+ v_{23}- v_{21}- v_{16}$$

$$\frac{d\left[ Sh-G \right]}{dt}= v_{18}+ v_{21}- v_{22}$$

$$\frac{d\left[ PLCg \right]}{dt}= v_{8}- v_{5}$$

$$\frac{d\left[ PLCgP \right]}{dt}= v_{7}- v_{8}- v_{25}$$

$$\frac{d\left[ PLCgP-I \right]}{dt}= v_{25}$$

$$\frac{d\left[ Grb \right]}{dt}= v_{12}- v_{9}- v_{17}- v_{21}$$

$$\frac{d\left[ Shc \right]}{dt}= v_{16}- v_{13}$$

$$\frac{d\left[ SOS \right]}{dt}= v_{12}- v_{10}- v_{19}- v_{22}$$
