# Supplementary material for: Hybrid multiscale modeling and prediction of cancer cell behavior
Source: PLoS One. 2017 Aug 28;12(8):e0183810. doi: 10.1371/journal.pone.0183810 (PMC5573302; doi:10.1371/journal.pone.0183810)
Supplement: S2 Appendix — (DOCX) [file pone.0183810.s002.docx]

# S2 Appendix

**TNF Signaling pathways**: Kinetic equations comprising the computational model for TNF signaling [56].

$$\frac{dc_{1}}{dt} = -k_{1}. c_{1}. c_{2}+ k_{2}. c_{3}$$

$$\frac{dc_{2}}{dt} = -k_{1}. c_{1}. c_{2}+ k_{2}. c_{3}+ k_{17}. c_{18}+ k_{11}. c_{11}$$

$$\frac{dc_{3}}{dt} = k_{1}. c_{1}. c_{2}. k_{2}. c_{3}. k_{3}. c_{3}. c_{4}+ k_{4}. c_{5}$$

$$\frac{dc_{4}}{dt} = -k_{3}. c_{3}. c_{4}+ k_{4}. c_{5}+ k_{11}. c_{11}+ k_{20}. c_{21}$$

$$\frac{dc_{5}}{dt} = k_{3}. c_{3}. c_{4}. k_{4}. c_{5}. k_{5}. c_{5}. c_{6}+ k_{6}. c_{7}$$

$$\frac{dc_{6}}{dt} = -k_{5}. c_{5}. c_{6}+ k_{6}. c_{7}+ k_{11}. c_{11}+ k_{20}. c_{21}$$

$$\frac{dc_{7}}{dt} = k_{5}. c_{5}. c_{6}. k_{6}. c_{7}. k_{7}. c_{7}. c_{8}+ k_{8}. c_{9}$$

$$\frac{dc_{8}}{dt} = -k_{7}. c_{7}. c_{8}+ k_{8}. c_{9}+ k_{11}. c_{11}+ k_{20}. c_{21}$$

$$\frac{dc_{9}}{dt} = k_{7}. c_{7}. c_{8}. k_{8}. c_{9}. k_{9}. c_{9}. c_{10}+ k_{10}. c_{11}. k_{15}. c_{9}. c_{17}+ k_{16}. c_{18}$$

$$\frac{dc_{10}}{dt} = -k_{9}. c_{9}. c_{10}+ k_{10}. c_{11}+ k_{14}. c_{14}$$

$$\frac{dc_{11}}{dt} = k_{9}. c_{9}. c_{10}. k_{10}. c_{11}. k_{11}. c_{11}$$

$$\frac{dc_{12}}{dt} = -k_{12}. c_{12}. c_{13}+ k_{13}. c_{14}+ k_{11}. c_{11}$$

$$\frac{dc_{13}}{dt} = -k_{12}. c_{12}. c_{13}+ k_{13}. c_{14}+ k_{29}. c_{16}. c_{31}$$

$$\frac{dc_{14}}{dt} = k_{12}. c_{12}. c_{13}. k_{13}. c_{14}. k_{14}. c_{14}$$

$$\frac{dc_{15}}{dt} = k_{14}. c_{14}$$

$$\frac{dc_{16}}{dt} = k_{14}. c_{14}. k_{29}. c_{16}. c_{31}$$

$$\frac{dc_{17}}{c_{t}}= -k_{15}. c_{9}. c_{17}+ k_{16}. c_{18}+ k_{20}. c_{21}$$

$$\frac{dc_{18}}{dt} = k_{15}. c_{9}. c_{17}. k_{16}. c_{18}. k_{17}. c_{18}$$

$$\frac{dc_{19}}{c_{t}}= k_{17}. c_{18}. k_{18}. c_{19}. c_{20}+ k_{19}. c_{21}$$

$$\frac{dc_{20}}{dt} = -k_{18}. c_{19}. c_{20}+ k_{19}. c_{21}$$

$$\frac{dc_{21}}{dt} = k_{18}. c_{19}. c_{20}. k_{19}. c_{21}. k_{20}. c_{21}$$

$$\frac{dc_{22}}{dt} = k_{20}. c_{21}. k_{21}. c_{22}. c_{23}+ k_{22}. c_{24}+ k_{23}. c_{24}$$

$$\frac{dc_{23}}{dt} = -k_{21}. c_{22}. c_{23}+ k_{22}. c_{24}$$

$$\frac{dc_{24}}{dt} = k_{21}. c_{22}. c_{23}. k_{22}. c_{24}. k_{23}. c_{24}$$

$$\frac{dc_{25}}{dt} = k_{23}. c_{24}. k_{28}. c_{27}. c_{25}. k_{24}. c_{29}. c_{25}+ k_{25}. c_{30}+ k_{26}. c_{30}$$

$$\frac{dc_{26}}{dt} = k_{26}. c_{30}$$

$$\frac{dc_{27}}{dt} = p . c_{16}\left( t-\tau\right)- k_{28}. c_{27}. c_{25}$$

$$\frac{dc_{28}}{dt} = k_{28}. c_{27}. c_{25}$$

$$\frac{dc_{29}}{dt} = -k_{24}. c_{29}. c_{25}+ k_{25}. c_{30}$$

$$\frac{dc_{30}}{dt} = k_{24}. c_{29}. c_{25}. k_{25}. c_{30}. k_{26}. c_{30}$$

$$\frac{dc_{31}}{dt} = p . c_{16}\left( t-\tau\right)- k_{29}. c_{16}. c_{31}$$
