# Supplementary material for: Hybrid multiscale modeling and prediction of cancer cell behavior
Source: PLoS One. 2017 Aug 28;12(8):e0183810. doi: 10.1371/journal.pone.0183810 (PMC5573302; doi:10.1371/journal.pone.0183810)
Supplement: S3 Appendix — (DOCX) [file pone.0183810.s003.docx]

# S3 Appendix

**Model Parameters Estimation**: The values of parameters and thresholds of the model are presented in A and B Tables respectively. In equation 3 and equation 4, as the spatial step was fixed (20μm) and diffusion coefficient is 1.50×10^-7^ ≤ D_nutrient_ ≤ 6.70×10^-7^, step time has to be chosen to obtain a stable scheme for diffusion. Therefore, Fourier stability conditions are given by the diffusion number $R=\frac{D\times\Delta t}{{\Delta S}^{2}}<0.5$, which give us ∆t < 3s.

Table A. Model parameters used in our model.

| **Parameter** | **Value** | **Description** |
| --- | --- | --- |
| Simulation Step | **1** hour | - |
| ∆t | **1** s | Simulation time step |
| ∆S | **2×10** μm | Size of grid site |
| D_Oxygen_ | **1.67×10^-7^** cm^2^/s [48] | Oxygen diffusion coefficient |
| D_Glucose_ | **6.70×10^-7^** cm^2^/s [14] | Glucose diffusion coefficient |
| D_TNFα_ | **1.50×10^-7^** cm^2^/s (estimated) | TNFα diffusion coefficient |
| D_TGFα_ | **5.18×10^-7^** cm^2^/s [24] | TGFα diffusion coefficient |
| D_VEGF_ | **2.90×10^-7^** cm^2^/s [14] | VEGF diffusion coefficient |
| ${Pe}_{Oxygen}$ | **1×10^-3^** μm/s [59] | Oxygen permeability |
| ${Pe}_{Glucose}$ | **3×10^-1^** μm/s [14] | Glucose permeability |
| ${Pe}_{VEGF}$ | **1×10^-1^** μm/s [14] | VEGF permeability |
| $C_{Oxygen}^{art}$ | **5.56×10** μMolar (estimated) | Concentration of oxygen in arteriole |
| $C_{Glucose}^{art}$ | **57×10^3^** μMolar (estimated) | Concentration of glucose in arteriole |
| $C_{Oxygen}^{cap}$ | **4.17×10** μMolar (estimated) | Concentration of oxygen in capillary |
| $C_{Glucose}^{cap}$ | **57×10^3^** μMolar (estimated) | Concentration of glucose in capillary |
| $r_{art}$ | **3×10** μm (estimated) | Radius of arteriole |
| $r_{cap}$ | **1×10** μm [53] | Radius of capillary |
| $\omega_{VEGF}$ | **1×10^-2^** s^-1^ [60] | Constant variable |
| $\phi_{c}$ | **2×10^-2^** nMolar/s [15] | Constant variable |
| $\beta_{c}$ | **3.34×10^-3^** s^-1^ [48] | Constant variable |
| $\alpha_{n}$ | **1.67×10^-5^** s^-1^ [48] | Constant variable |

Table B. Model thresholds for used in our model.

| **Threshold Name** | **Value** |
| --- | --- |
| **Cancerous Cells Thresholds** | |
| Oxygen Threshold | $\left\{ \begin{aligned} \boldsymbol{1.175}If the cell is surrounded by more cancerous than healthy cells \\ \boldsymbol{3.525}If the cell is surrounded by more healthy than cancerous cells \end{aligned} \right.$ [53] |
| Glucose Dead Threshold | **8** μMolar [24] |
| Glucose Active Threshold | **16** μMolar [24] |
| Proliferation Time Delay Threshold | $\left\{ \begin{aligned} \boldsymbol{1}For selecting hypoxia phenotype \\ \boldsymbol{8} For selecting necrosis phenotype \end{aligned} \right.$ [55] |
| Hypoxia Threshold | $\left\{ \begin{aligned} \boldsymbol{1}For selecting hypoxia phenotype \\ \boldsymbol{67} For selecting necrosis phenotype \end{aligned} \right.$[50] |
| TNFα Threshold Range | (0.6,6) [56] |
| **Healthy Cells Thresholds** | |
| Oxygen Threshold | $\left\{ \begin{aligned} \boldsymbol{35.25}If the cell is surrounded by more cancerous than healthy cells \\ \boldsymbol{352.5}If the cell is surrounded by more healthy than cancerous cells \end{aligned} \right.$ [53] |
| Glucose Dead Threshold | **8** μMolar [24] |
| Glucose Active Threshold | **16** μMolar [24] |
| Cell Division Threshold | 50 (estimated) |
| Proliferation Time Delay Threshold | $\left\{ \begin{aligned} \boldsymbol{1}For selecting hypoxia phenotype \\ \boldsymbol{2} For selecting apoptosis phenotype \end{aligned} \right.$ [55] |
| Hypoxia Threshold | $\left\{ \begin{aligned} \boldsymbol{1}For selecting hypoxia phenotype \\ \boldsymbol{2} For selecting apoptosis phenotype \end{aligned} \right.$[50] |
| TNFα Threshold Range | (0.6,6) [56] |
| **Vessel Cells Thresholds** | |
| ψ | 18 [61] |
