# Supplementary material for: Hybrid multiscale modeling and prediction of cancer cell behavior
Source: PLoS One. 2017 Aug 28;12(8):e0183810. doi: 10.1371/journal.pone.0183810 (PMC5573302; doi:10.1371/journal.pone.0183810)
Supplement: S4 Appendix — (DOCX) [file pone.0183810.s004.docx]

# S4 Appendix

The Figs A and B shows a realization of a 40×40×40 domain with a cube of tumor cells implanted in healthy tissue with eight straight initial vessels. Healthy cells are always eliminated and the tumor invades the entire domain. Fig C shows a representative profile of the spatial distribution of nutrient concentration. It is seen that during the initial growth stages, a core of necrotic cells appears at the center of tumor surrounded by proliferating cells.


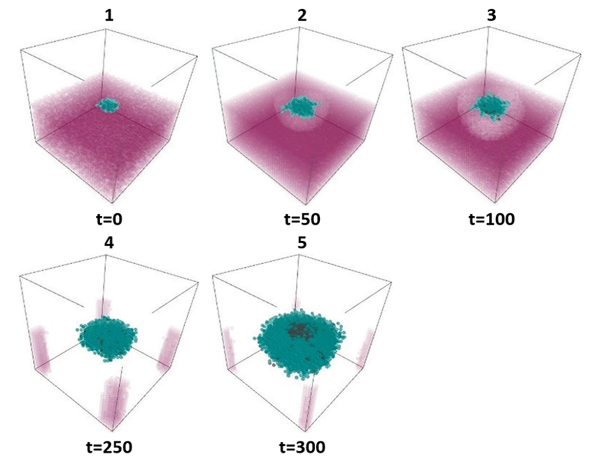


Fig A. Graphical visualization of tumor morphologies at different time from vertical view.


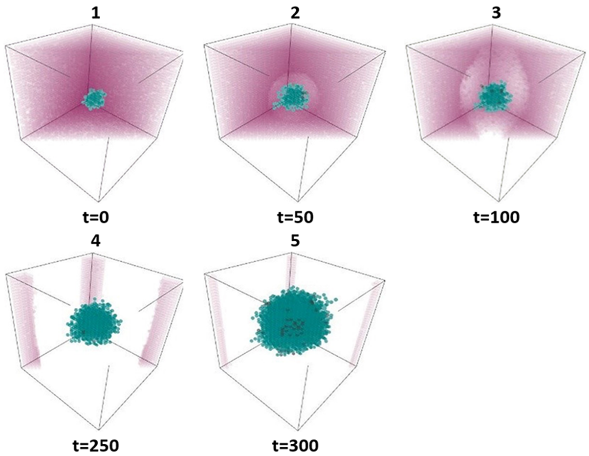


Fig B. Graphical visualization of tumor morphologies at different time from horizontal view.


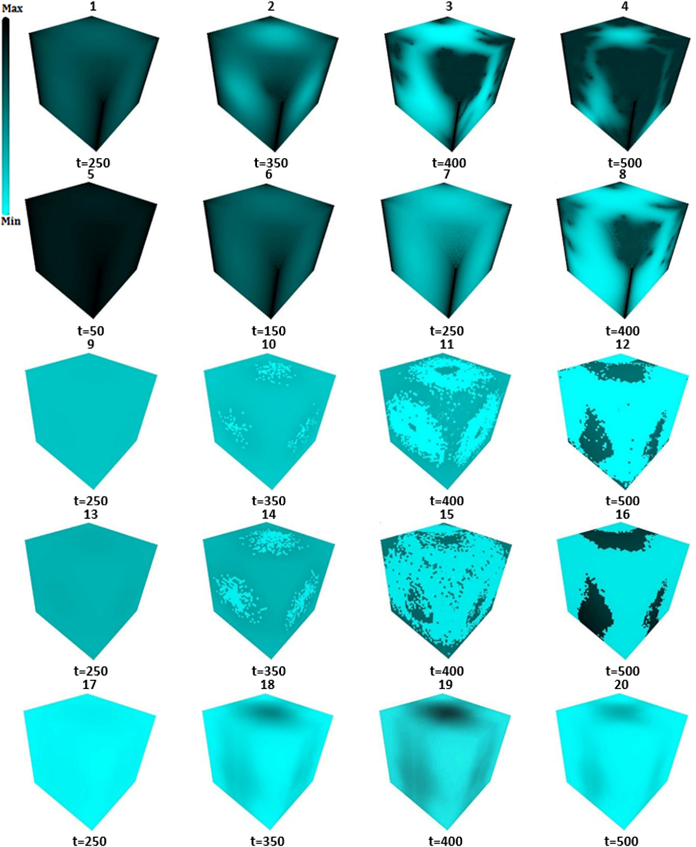


Fig C. Showing the concentration profile of nutrient supplied from the vasculature. The first to fifth rows of figures show Glucose, Oxygen, TNF, TGF and VEGF distribution over four time steps.
